# Dataset of Inter and intramuscular variability of stiffness in paretic individuals during prone and standing positions

**DOI:** 10.1016/j.dib.2024.110190

**Published:** 2024-02-11

**Authors:** Kalthoum Belghith, Mustapha Zidi, Jean Michel Fedele, Rayan Bou-Serhal, Wael Maktouf

**Affiliations:** a Bioengineering, Tissues and Neuroplasticity, UR 7377, University of Paris-Est Creteil, Faculty of Medicine, Creteil, France; b CLINEA clinics, Clinique du Parc de Belleville, Paris, France

**Keywords:** Skeletal muscle, Spasticity, Shear wave elastography, Plantar flexors, Data

## Abstract

Several studies have investigated muscle rigidity using SWE. However, the assessments may not consider the most affected regions within the same muscle tissue nor the intramuscular variability of rigidity between muscles of the same muscle group, e.g., plantar flexors. The data presented in this article aimed to explore the inter–and intramuscular variability of plantar flexors stiffness during prone and standing positions at different muscle lengths in healthy and paretic individuals. Shear wave ultrasound images were acquired for the three plantar flexor muscles (gastrocnemius medialis [GM], gastrocnemius lateralis [GL], and soleus [SOL]) in two positions: prone and standing. The imaging was conducted at various dorsiflexion angles (0°, 10°, and 20°), and measurements were taken at different proximo-distal regions within each muscle. This data set allowed us to highlight the impact of stroke on mechanical properties that varies depending on whether ankle muscles are in an active or passive state during dorsiflexion. Additionally, the modification of the ankle muscle state influences the distribution of stiffness both within and between the plantar flexors.

Specifications Table

**Table:** 

Subject	*Biomechanics and neuroscience*
Specific subject area	*Shear Wave Elastography (SWE) imaging*
Data format	*Raw, Analyzed*
Type of data	*Images and DICOM files*
Data collection	An Aixplorer^Ⓡ^ ultrasound scanner (Supersonic Imagine, version 6.1, Aix-enProvence, France) in Shear Wave mode (musculoskeletal preset, penetration mode, scale: 200 kPa) was used to quantify muscle stiffness in plantar flexors (GM, GL, and SOL). During the evaluations, two ultrasound probes were used: probe 1 (4 – 15 MHz, SL15-4; SuperSonic Imagine, Aix-en-Provence, France) for the GM and GL, and probe 2 (2 – 10 MHz, SL10-2; SuperSonic Imagine, Aix-en-Provence, France) for the SOL.
Data source location	Bioengineering, Tissues and Neuroplasticity, UR 7377, University of Paris-Est Creteil, Faculty of Medicine, Creteil, France CLINEA group, Clinique du Parc de Belleville, Paris, France
Data accessibility	Repository name: *Belghith, Kalthoum; Mustapha, Zidi; Bou Serhal, Rayan; Fedele, Jean Michele; Maktouf, WAEL (2023), “Data set of Spatial distribution of stiffness between and within muscles in paretic and healthy individuals during prone and standing positions”, Mendeley Data, V1* Data identification number: doi: 10.17632/7fsmmsh5gh.1 Direct URL to data: https://data.mendeley.com/datasets/7fsmmsh5gh/1
Related research article	Belghith, K., Zidi, M., Fedele, J. M., Serhal, R. B., & Maktouf, W. (2023). Spatial distribution of stiffness between and within muscles in paretic and healthy individuals during prone and standing positions. Journal of Biomechanics, 111838. https://doi.org/10.1016/j.jbiomech.2023.111838

## 1 Value of the Data

• To the best of our knowledge, this dataset comprises the pioneering SWE images capturing stiffness in both paretic and healthy individuals during the standing position.
• The dataset accentuates the importance of considering the spatial distribution of stiffness within and between muscles.
• The dataset sheds light on the impact of muscle position, providing insights for a comprehensive understanding of stiffness distribution during plantar flexors stretching.
• The dataset underscores that the distribution of muscle stiffness varies based on the engagement of both the contractile (active condition) and elastic (passive condition) components.
• The dataset provides valuable insights for a comprehensive understanding of the influence of stroke on ankle muscle stiffness and its distribution within and between plantar flexors.
• Muscle stiffness distribution varies depending on the involvement of the contractile and elastic components during movement.

## 2 Background

Stroke injury induces muscle contracture, characterized by an increase in muscle stiffness, potentially leading to a decreased range of motion and mobility. Various studies have utilized SWE, to investigate the mechanical adaptation of paretic muscles. Notably, these studies have consistently demonstrated elevated muscle stiffness in the paretic muscle [[1], [2]]. However, assessments in these studies may not comprehensively consider the most affected regions within the same muscle tissue or account for intramuscular variability of stiffness [3].

Furthermore, the primary objective of post-stroke rehabilitation is to restore walking autonomy and prevent spasticity, often achieved through a self-stretching program in a quiet standing position. Remarkably, no prior studies have specifically explored plantar flexor stiffness during a quiet standing position or investigated the distribution of stiffness within these muscles [3].

This data article aims to elucidate the process of measuring ankle muscle stiffness using SWE and guide clinicians in analysing data. The information provided can be particularly beneficial for clinicians employing the same technique for diagnosing stiffness in stroke survivors or individuals with other neurological pathologies.

## 3 Data description

### 3.1 Data collecting

This data article presents inter- and intramuscular variability of stiffness among paretic and healthy plantar flexors during prone and standing positions. SWE images were collected and stored in a DICOM format. The SWE dataset is thoughtfully organized into two distinct classes: images captured in the prone position and those taken during the quiet standing position. Within each class, 24 images were curated, featuring 8 images for each specified position (0°, 10°, and 20° of dorsiflexion).

### 3.2 Processing

The SWE technique can be described in three steps. First, shear waves are generated within the muscle. Second, generated shear waves propagate through the adjacent tissues, causing shear displacements, which are calculated using a speckle-tracking algorithm. Third, tissue displacement maps are used to calculate shear-wave velocity (Vs, m/s) in each pixel of the map. Then, muscle stiffness was estimated from the calculation of the shear modulus (µ) assuming that the mechanical behavior is elastic, isotropic, and incompressible as follows: **µ = ρ.Vs²**, where ρ is the muscle mass density (1000 kg/m^3^).

**Fig. 1:**
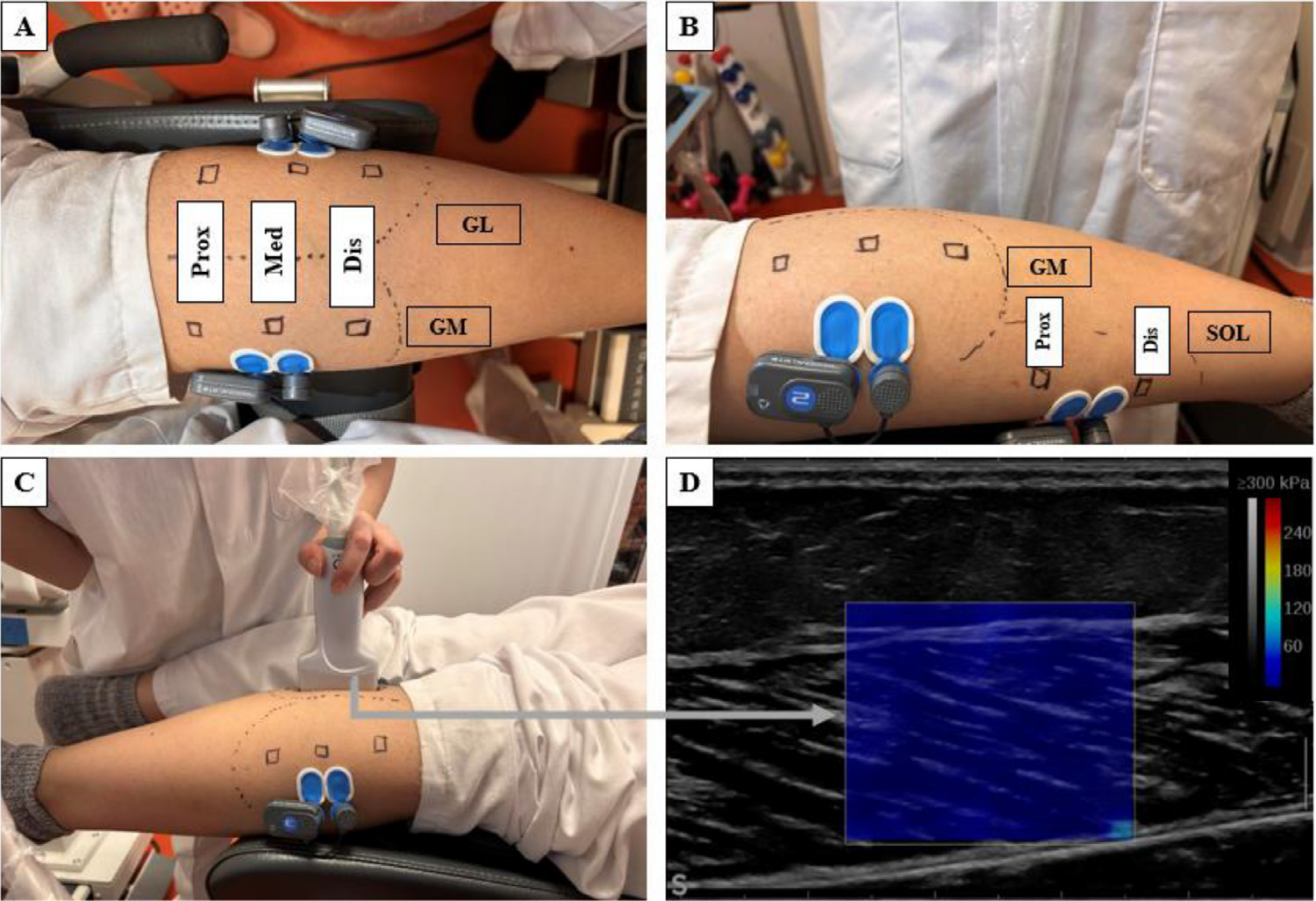
Experimental set-up used to determine muscle stiffness in prone position. (A) Identification of gastrocnemius medialis (GM) and lateralis (GL) regions and the placement of EMG electrodes. (B) Identification of soleus regions (SOL) and the placement of EMG electrodes for SOL. (C) Probe positioning to determine muscle stiffness. (D) Shear modulus (µ) maps of a representative healthy gastrocnemii medialis in medial region during the passive mobilization of the ankle joint. PROX: proximal region, MED: medial region, DIS: distal region.

**Fig. 2:**
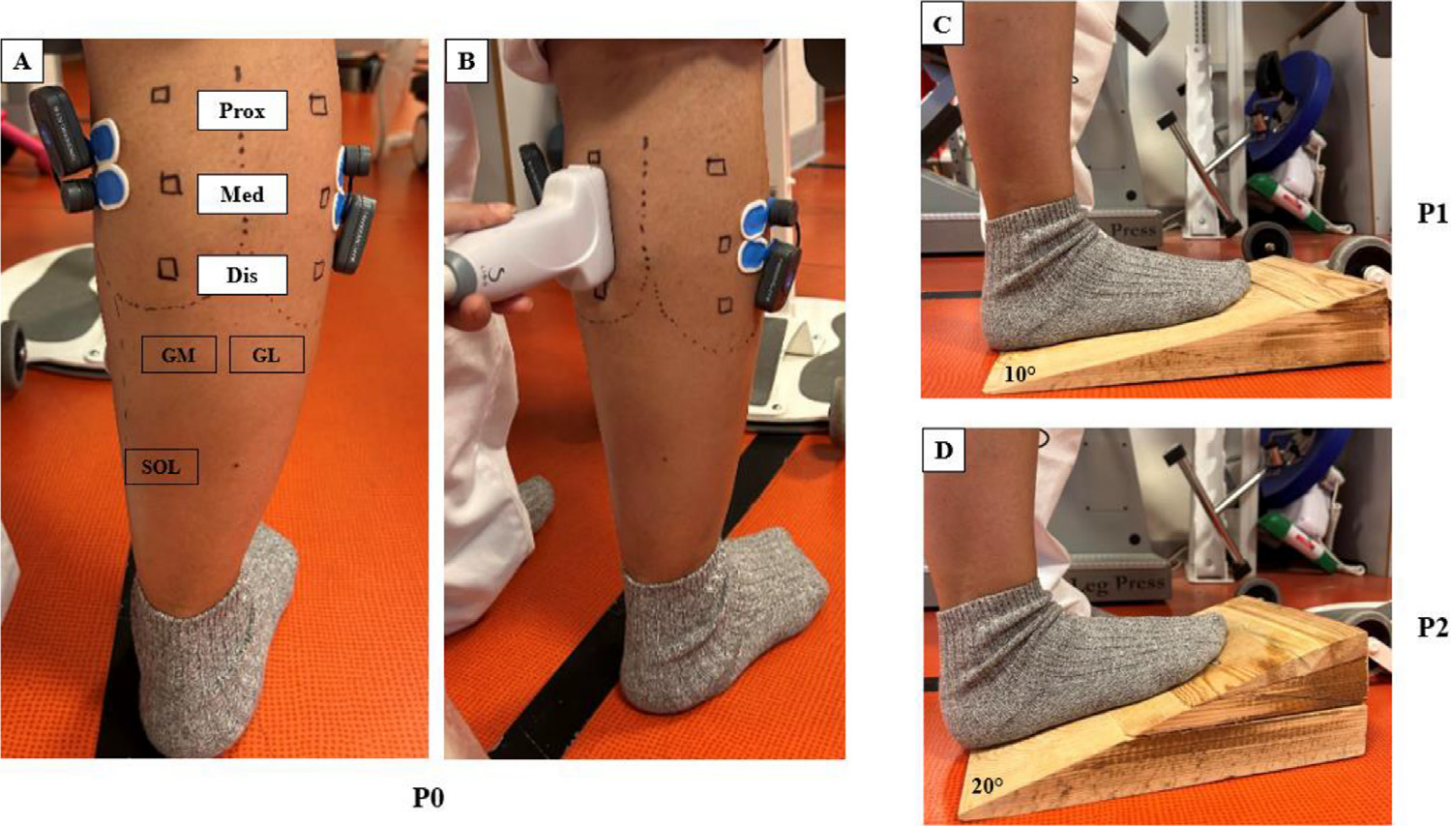
Experimental set-up used to determine muscle stiffness in the quiet standing position. (A) Identification of gastrocnemius medialis (GM) and lateralis (GL), and soleus (SOL) regions and the placement of EMG electrodes. (B) Probe positioning to determine muscle stiffness. (C) Wooden wedges for 10° of dorsiflexion used for assessment. (D) Wooden wedges for 20° of dorsiflexion used for assessment. P0: without wooden wedge, P1: wooden wedge at 10° to the floor, P2: wooden wedge at a 20° to the floor.

Based on the anatomical landmarks and the experimental protocol of Le sant et al. [1], three regions were identified for the GM and GL (proximal [PROX], medial [MED] and distal [DIS]) and two regions for the SOL (PROX and DIS) using B-mode elastography. The probe locations for each region were marked on the skin using waterproof ink. Finally, the Vs was evaluated in these eight regions for each experimental protocol assessment, as illustrated in Fig. 1 (prone position) and Fig. 2 (quiet standing position).

Dataset were collected and then analyzed using Matlab software (Matlab R2021a, MathWorks, Natick, USA). Ultrasound images were exported from Aixplorer's software. Image processing converted each pixel of the color map into a shear modulus based on the recorded scale.

**Fig. 3:**
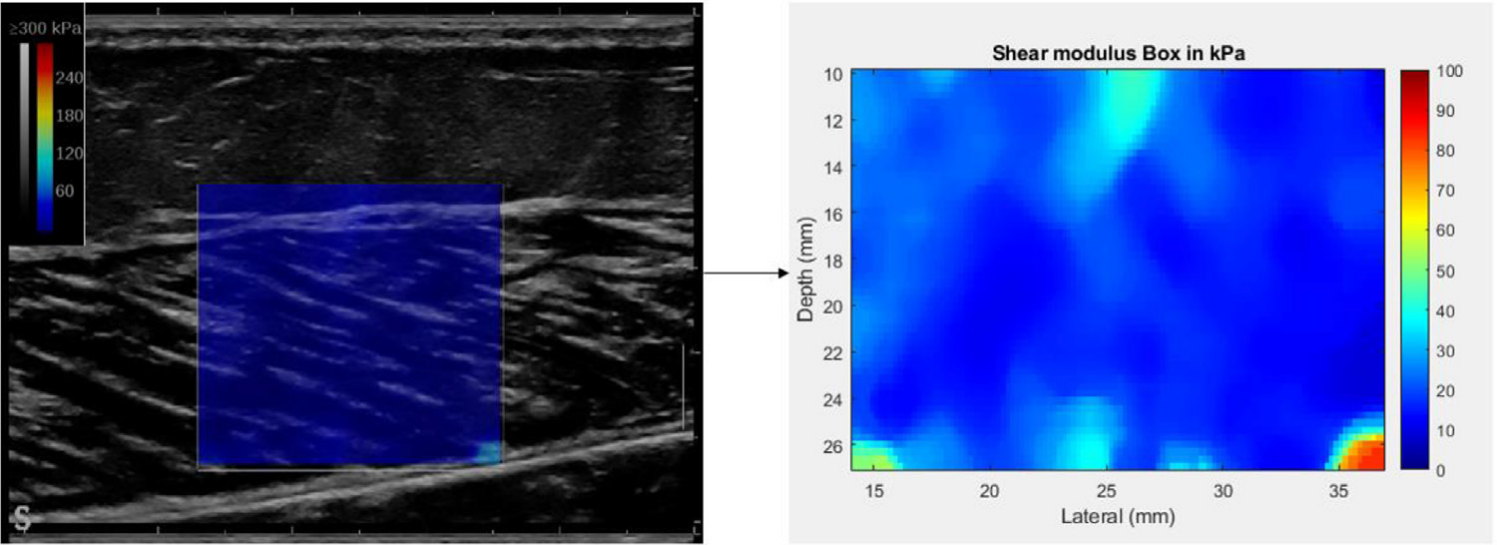
Shear modulus map of a representative GM med analyzed by MATLAB.

Mean shear modulus values were calculated in a 15 × 15 mm^2^ region of interest in different regions of each muscle fascicular area (Fig 3).

## 4 Experimental Design, Materials and Methods

### 4.1 Trial design

This investigation is a cross-sectional analytical study, extending over a period of 3 months. The study was carried out in three phases: an initial recruitment phase of 1-3 weeks, followed by a screening period lasting 1-2 weeks, and finally a 7-week experimental testing period. Each experimental session, which ranged between 60 to 75 minutes, comprised of three key assessments: (1) determination of the maximum range of motion (ROM) and maximum voluntary contraction (MVC) of ankle muscles [4], (2) evaluation of the shear modulus during prone position (3) evaluation of the shear modulus during standing position.

### 4.2 Population

We initially approached 35 individuals from at the Clinic of Parc de Belleville, a neurological rehabilitation clinic located in Paris. However, 7 of these individuals did not meet the established inclusion criteria 7 and were consequently excluded. Additionally, two potential participants chose not to partake in this study. As a result, the final participant pool consisted of 28 individuals, who were then systematically divided into two distinct groups. The control group (CG) consisted of 14 healthy individuals with no history of neurological or muscular disorders (n = 14; age 43.9 ± 9.6 years; body mass index [BMI] = 24.5 ± 2.5 kg/m^2^). The stroke survivor group (SSG) consisted of 14 stroke survivors with spastic hemiparesis (SSG; n = 14; age 43.9 ± 9.6 years; BMI = 24.5 ± 2.5 kg/m^2^). The exclusion criteria for this study included the presence of other neurological conditions, ankle pathology, metal implants in the lower extremities, and recent surgery in the lower extremities or lumbar spine. Additionally, participants in the stroke group had to meet the following inclusion criteria: a diagnosis of hemispheric stroke occurring more than 6 months prior to the study, and exclusion criteria of brainstem or cerebellar stroke, botulinum toxin therapy within the previous 6 months, and severe contractures that limited the ability to achieve neutral dorsi-plantarflexion (0°) [2].

### 4.3 Outcomes

For the prone position, the participants were placed in a prone position on the isokinetic machine and instructed to completely relax. To ensure consistency in leg position and minimize the involvement of unmonitored muscles, the ankle was securely fixed in a relaxed position within the isokinetic dynamometer. Starting from the neutral position (0°), the ankle joint underwent passive mobilization, moving from plantar flexion to dorsal flexion at a slow angular velocity of 2°/s. This was done to assess the maximal range of motion, representing the stretch range before reaching the pain threshold. Participants were instructed to maintain maximum relaxation and to cease ankle rotation when they felt the maximum tolerated stretch. Two positions in dorsal flexion were identified from the neutral position (P0 = 0°): P1 = 10° and P2 = 20°. For each position, the SWE measurement was conducted twice in each pre-determined region, in a randomized order, with a 1-minute rest period between measurements. 8 maps of Vs were recorded for each position for further analysis. For the quiet standing position, the participants were standing with their hands holding onto a support. With the knee fully extended, and the leg perpendicular to the floor. SWE and EMG measurements were conducted both with and without the placement of wooden wedges under the foot. Tests were performed barefoot in three distinct positions: P0 (without wooden wedge), P1 (with a wooden wedge inclined at 10° to the floor), and P2 (with a wooden wedge inclined at 20° to the floor). In each position, SWE measurements were taken twice in pre-determined regions, following a randomized order, and separated by a 1-minute rest period. 8 maps of Vs were recorded for each position for further analysis.

## Limitations

Ultrasound shear wave elastography precisely gauges shear wave speed and its corresponding shear modulus along the principal axis of the probe, specifically along the transverse axis of the imaging plane, rather than aligning with the muscle fiber/fascicle direction. This distinction is crucial, given the well-established understanding that variations in muscular architecture, particularly in terms of pennation, can influence both shear wave speed and shear modulus. However, for this type of muscle, it has been demonstrated a relative reproducibility of stiffness measurements [5].

## Ethics statement

Ethical approval: All procedures performed in studies involving human participants received Institutional Ethics Committee approval (CPP 2022–038 = 000117) and were in accordance with the 1964 Helsinki declaration.

Informed consent: persons provided their written informed consent to trial participation and data publication.

## CRediT authorship contribution statement

**Kalthoum Belghith:** Writing – original draft, Visualization, Supervision, Software, Resources, Methodology, Investigation, Formal analysis, Data curation, Conceptualization. **Mustapha Zidi:** Writing – review & editing, Visualization, Validation, Supervision, Conceptualization. **Jean Michel Fedele:** Investigation, Project administration, Resources, Funding acquisition. **Rayan Bou-Serhal:** Resources, Project administration, Data curation. **Wael Maktouf:** Visualization, Validation, Supervision, Methodology, Investigation, Funding acquisition, Conceptualization.

## Data Availability

Data set of Spatial distribution of stiffness between and within muscles in paretic and healthy individuals during prone and standing positions (Original data) (Mendeley Data).

## References

[1] le Sant G., Gross R., Hug F., Nordez A. (2019). Influence of low muscle activation levels on the ankle torque and muscle shear modulus during plantar flexor stretching. J. Biomech..

[2] Huang M., Miller T., Fu S.N., Ying M.T.C., Pang M.Y.C. (2022). Structural and passive mechanical properties of the medial gastrocnemius muscle in ambulatory individuals with chronic stroke. Clin. Biomech..

[3] Belghith K., Zidi M., Fedele J.M., Bou Serhal R., Maktouf W. (2023). Spatial distribution of stiffness between and within muscles in paretic and healthy individuals during prone and standing positions. J. Biomech..

[4] Maktouf W., Durand S., Boyas S., Pouliquen C., Beaune B. (2018). Combined effects of aging and obesity on postural control, muscle activity and maximal voluntary force of muscles mobilizing ankle joint. J. Biomech..

[5] Miyamoto N., Hirata K., Kanehisa H., Yoshitake Y. (2015). Validity of measurement of shear modulus by ultrasound shear wave elastography in human pennate muscle. PloS One.

